# Proceedings of the Boston Society for Medical Observation

**Published:** 1879-03

**Authors:** A. M. Sumner

**Affiliations:** Secretary


### PROCEEDINGS OF THE BOSTON SOCIETY FOR MEDICAL OBSERVATION.

A. M. SUMNER, M,D., SECRETARY.

---

December 16, 1878. *Symmetrical Gangrene of the Extremities.*—Dr. J. Collins Warren read a paper on Symmetrical Gangrene of the Extremities, which was published in the JOURNAL, January 16th.

Dr. T. B. Curtis wished to notice in this connection how little attention is paid to French sources of investigation. While English and German authorities are as a rule pretty well ransacked, the French sources are overlooked.

Dr. F. W. Draper asked if the terms local syncope and local asphyxia were not simply appearances of the parts affected rather than true symptoms.

Dr. Warren answered that the absence of blood was termed syncope, while local asphyxia was an expression drawn from a term designating a condition popularly called "black in the face,"—black without actually becoming gangrenous.

Dr. Ingalls inquired if in the cases reported by Dr. Weir Mitchell the first symptoms of local syncope were alluded to or spoken of.

Dr. Warren said that in some of the cases they were alluded to, but in others they were passed by unnoticed. Where they had arrived at true gangrene the patients possibly had overlooked the earlier symptoms.

Dr. J. J. Putnam remarked that he had seen one marked case of local asphyxia, such as Dr. Warren had spoken of. It was in the person of a young man accustomed to out-of-door life, and otherwise perfectly healthy. The affection had already lasted a year or two. Whenever he went out of doors, even in summer, the hands, especially those portions supplied by the ulnar nerves, would turn white, afterwards again becoming of normal color after passing through the blue stage. It was also noticeable that the parts were painful, not as Dr. Warren had stated during the algid stage, but while the normal color was coming back. Vulpian raises the question whether these changes are due to disturbances of function of the local peripheral ganglia controlling the circulation, or to disturbances in the action of the vaso-motor centres, and considers the former explanation as a possible one for some cases. Dr. Putnam thought that both causes might exist, it being probable that the local and the central ganglia act and react on each other both in health and disease. In this connection he said that he had seen a case where, in consequence of injury of the brachial plexus, the whole hand had become cyanotic, while all the nerves of the arm were paralyzed. After a time the ulnar nerve, with its cerebral and spinal nuclei, recoverd its control over the muscles supplied by it, and at the same time presumably over the blood-vessels of the outer portion of the hand. In consequence of this the color of that part of the hand became again natural, standing in marked contrast to that of the rest of the hand. Dr. Putnam's theory was that this resulted from a resumption of the controlling or inhibitory action which the spinal centres normally exercised upon the peripheral vaso-motor ganglia. Dr. Putnam also agreed with Vulpian that there seemed to be no reason for assuming contraction of the veins, besides that of the arteries, to explain these local asphxias.

Dr. C. P. Putnam remarked that he had seen a case of the so-called syncope and asphxia of the fingers in a young lady of good health, where it had apparently been due to cold morning baths, as it had not occurred since the baths were given up. In the first stage the affected finger, generally the middle or third finger on either hand, became white and the skin wrinkled; soon after became intensely blue or purple, and finally recovered its color in an hour or two, with considerable tingling. The patient always found that the quickest way to get rid of an attack was to heat the back before a fire or by hot air from a furnace, though it seems likely that this was only because the whole body was most easily healed in this way.

Dr. C. H. Williams asked whether, as the ophthalmoscope showed diminution of the calibre, there was any deficiency of vision, and whether it was permanent.

Dr. Warren replied that there was intermittent disturbance, but did not think that the disturbance was permanent.

Dr. Hildreth inquired if there was any material difference of color, in these cases, from senile gangrene.

Dr. Warren said that authorities undertake to make out a peculiar shade in the local asphyxia, but after the gangrene appears there is very little difference. Dr. Warren thought that there was a difference in this respect between a case of embolic gangrene and the case reported, as seen by him at the same time.

*Scarlatina*—Dr. Minot remarked that he had seen a case which might be of interest. He was called to see an infant with scarlatina. Two months before it was born the mother and two children passed through a very severe attack of the same disease. It would certainly seem as though the infant was protected, but such was not the case.

*Jaborandi*—Dr. Webber wishes to state his experience with Metcalf's preparation of jaborandi. He had given it in twelve cases with no effect, and in one case with slight effect. He then had an infusion made with the dried leaves at the City Hospital, and obtained a marked effect in most cases.

Dr. Minot had had the same experience, but when he ordered three or four drachms of the leaves to a pint of water, and had the mixture boiled until it measured half a pint, and drunk hot, he had obtained marked effect.

